# Effects of Aging, Word Frequency, and Text Stimulus Quality on Reading Across the Adult Lifespan: Evidence From Eye Movements

**DOI:** 10.1037/xlm0000543

**Published:** 2018-04-19

**Authors:** Kayleigh L. Warrington, Victoria A. McGowan, Kevin B. Paterson, Sarah J. White

**Affiliations:** 1 Department of Neuroscience, Psychology and Behaviour, University of Leicester

**Keywords:** eye movements, stimulus quality, reading, aging

## Abstract

Reductions in stimulus quality may disrupt the reading performance of older adults more when compared with young adults because of sensory declines that begin early in middle age. However, few studies have investigated adult age differences in the effects of stimulus quality on reading, and none have examined how this affects lexical processing and eye movement control. Accordingly, we report two experiments that examine the effects of reduced stimulus quality on the eye movements of young (18–24 years), middle-aged (41–51 years), and older (65+ years) adult readers. In Experiment 1, participants read sentences that contained a high- or low-frequency critical word and that were presented normally or with contrast reduced so that words appeared faint. Experiment 2 further investigated effects of reduced stimulus quality using a gaze-contingent technique to present upcoming text normally or with contrast reduced. Typical patterns of age-related reading difficulty (e.g., slower reading, more regressions) were observed in both experiments. In addition, eye movements were disrupted more for older than younger adults when all text (Experiment 1) or just upcoming text (Experiment 2) appeared faint. Moreover, there was an interaction between stimulus quality and word frequency (Experiment 1), such that readers fixated faint low-frequency words for disproportionately longer. Crucially, this effect was similar across all age groups. Thus, although older readers suffer more from reduced stimulus quality, this additional difficulty primarily affects their visual processing of text. These findings have important implications for understanding the role of stimulus quality on reading behavior across the lifespan.

Numerous studies with young adult participants show that words are more difficult to recognize when stimulus quality is reduced by presenting text in lower contrast so that words appear faint (e.g., Becker & Killion, 1977). Some studies have also investigated the effects of reduced visual contrast on the reading performance of individuals with visual impairments (e.g., Legge, Rubin, Pelli, & Schleske, 1985). However, few studies have investigated effects for typical older adult readers (e.g., Mitzner & Rogers, 2006), although it is well-established that sensory declines that begin early in middle age and manifest more severely in older age might cause readers to also experience greater difficulty. Indeed, older adults commonly self-report difficulties associated with contrast in visual quality-of-life assessments, such as problems seeing in dim light and distinguishing between dark colors (Kosnik, Winslow, Kline, Rasinski, & Sekuler, 1988). Consequently, as text encountered in everyday reading can vary substantially in contrast (e.g., resulting from poor print or display quality) and reading often occurs in suboptimal luminance (Charness & Dijkstra, 1999), changes in visual contrast may have important consequences for older adults’ reading performance. Measures of eye movements provide detailed information about component processes in normal reading, including those involved in eye movement control and the identification of words (Liversedge & Findlay, 2000; Rayner, 1998, 2009). Accordingly, we used measures of eye movements during reading to shed light on adult age differences in the effects of stimulus quality on reading performance, and specifically effects of stimulus quality on lexical processing.

## Adult Age Differences in Reading

Compared with young adults (aged 18–30 years), healthy older adults (aged 65+) typically experience greater reading difficulty and thus read more slowly despite normal levels of comprehension (Kemper, McDowd, & Kramer, 2006; Rayner, Reichle, Stroud, Williams, & Pollatsek, 2006; Stine-Morrow, Loveless, & Soederberg, 1996). In particular, older readers show a pattern of eye movement behavior that includes more and longer fixations (pauses of eyes) and more regressions (backward eye movements to earlier parts of the text). In addition, older adults often make longer forward eye movements (saccades) because of a greater likelihood of skipping past words without fixating them (e.g., Kliegl, Grabner, Rolfs, & Engbert, 2004; McGowan, White, Jordan, & Paterson, 2014; McGowan, White, & Paterson, 2015; Rayner, Liversedge, & White, 2006; Warrington, White, & Paterson, 2018; but see Choi, Lowder, Ferreira, Swaab, & Henderson, 2017; Whitford & Titone, 2016, 2017). This has led some researchers to argue that these differences in reading behavior reflect the adoption of a “risky” strategy, whereby older readers compensate for a slowdown in processing by inferring the identities of upcoming words based on only partial word information (Rayner, Castelhano, & Yang, 2009; Rayner et al., 2006). This more risky reading strategy results in longer forward saccades but also more frequent regressions to resample words when these guesses prove incorrect. However, this characterization of older adults’ reading behavior is currently a matter of debate (Choi et al., 2017). An alternative account argues that older adults may not adjust saccade programs as flexibly as young adults in response to local processing difficulties (see Risse & Kliegl, 2011; Wotschack & Kliegl, 2013).

Presently, it is not known when during the adult life span adult age differences in reading emerge. Gaining a greater understanding of when these differences emerge may help to shed light on the role of specific visual factors on reading performance. Some studies hint at a slowdown in reading for those in middle age (e.g., 35–59 years; Calabrèse et al., 2016; Soederberg Miller, 2009; see also Teramoto, Tao, Sekiyama, & Mori, 2012), although none have employed eye movement methods to examine these differences in detail. An important consideration in the current study, therefore, was to establish at what point during the adult life span low-contrast text becomes problematic for reading.

## Text Stimulus Quality

Eye movement studies with young adult readers have shown longer reading times for faint compared with normally presented text, both for entire sentences (Hohenstein & Kliegl, 2014; Jainta, Nikolova, & Liversedge, 2017; Liu, Li, & Han, 2015; White & Staub, 2012) and words within sentences (Drieghe, 2008; Glaholt, Rayner, & Reingold, 2014; Reingold & Rayner, 2006; Sheridan & Reingold, 2013; Wang & Inhoff, 2010; White & Staub, 2012). However, very few studies have examined effects of text stimulus quality on reading behavior for older adults (Mitzner & Rogers, 2006; see also Madden, 1988; Speranza, Daneman, & Schneider, 2000), and no studies have carried out a detailed assessment of effects of stimulus quality on eye movement behavior during reading for middle-aged and older adults. Given the loss of sensitivity to fine visual detail (such as letter features or individual letters; Crassini, Brown, & Bowman, 1988; Elliott, Whitaker, & MacVeigh, 1990; Owsley, Sekuler, & Siemsen, 1983) and reductions in contrast sensitivity (Crassini et al., 1988; Laitinen et al., 2005) that occur naturally in older adulthood, it is reasonable to speculate that high stimulus quality may be especially important for older adults. Indeed, a study by Mitzner and Rogers (2006), in which young and older adults read high-, medium-, and low-contrast sentences presented word-by-word, showed larger effects of text contrast on reading times for older adults compared with younger adults. Additionally, studies employing methods other than contrast reduction to reduce stimulus quality have also shown greater effects of text quality for older than young adults. Madden (1988) found that response times in a lexical decision task increased more for older than for young adults when asterisks were placed between adjacent letters, and Paterson, McGowan, and Jordan (2013a, 2013b; see also Jordan, McGowan, & Paterson, 2014) found that filtering the spatial frequency content of text affected young and older adults differently, with older adults experiencing greater reading difficulty than young adults when text lacked its normal full complement of spatial frequencies. Crucially, as many of the changes in visual abilities (including changes in contrast sensitivity) that older adults experience begin in middle age (40–50 years) and become more pronounced with older age (Owsley et al., 1983; Owsley, 2011; Schefrin, Tregear, Harvey, & Werner, 1999), poor stimulus quality also may be more detrimental for middle-aged readers compared with younger adult readers. This issue will be explored in the current study.

## Lexical Processing

Effects of word frequency on eye movement behavior during reading provide an index of lexical processing difficulty (Inhoff & Rayner, 1986; Rayner, Sereno, & Raney, 1996). Words that occur frequently in the language (e.g., *room*) are more likely to be skipped and have shorter reading times compared with words that occur less frequently (e.g., *crib*). Interestingly, older adults have sometimes been shown to produce larger word frequency effects than young adults, by making disproportionately longer fixations on words that have a lower frequency of written usage (Kliegl et al., 2004; McGowan et al., 2015; Rayner et al., 2006; Whitford & Titone, 2017; though note that this effect is often small and has not been found consistently across all studies: McGowan et al., 2014; Rayner, Yang, Schuett, & Slattery, 2013). Such results are in line with the possibility that lexical processing of words may be more difficult for older compared with younger adults. Crucially, in the present study, we further examine how stimulus quality affects word recognition processes for readers across the adult life span.

There has also been long-standing interest in how manipulations of text stimulus quality affect word recognition. A particular concern is to establish whether reductions in stimulus quality affect only the early encoding of visual and orthographic features or also the lexical processing of words (e.g., Becker & Killion, 1977). Additive effects of contrast and word frequency might indicate that these variables influence separate processing stages, whereas interactive effects might suggest they influence a common stage (Sternberg, 1969; but see McClelland, 1979). The effects of stimulus quality on lexical processing for young adults have been shown to depend on the specific task requirements (Yap & Balota, 2007; Yap, Balota, Tse, & Besner, 2008). Additive effects have been shown in lexical decision tasks (Balota & Abrams, 1995; Becker & Killion, 1977; Plourde & Besner, 1997; Stanners, Jastrzembski, & Westbrook, 1975; Yap & Balota, 2007; but see Balota, Aschenbrenner, & Yap, 2013), and interactive effects have been shown for naming and sematic categorization tasks (O’Malley, Reynolds, & Besner, 2007; Yap & Balota, 2007). Studies of eye movements during sentence reading have also shown interactive patterns of results when the stimulus quality and lexical frequency of a single critical word within a sentence is manipulated. Sheridan and Reingold (2013) showed that initial fixation durations on critical words produced an interactive pattern of effects, with larger word frequency effects for faint words compared with normally presented words. However, additive effects have been shown for studies for which the stimulus quality of the entire sentence was manipulated (Jainta et al., 2017; Liu et al., 2015).

To summarize, studies of effects of stimulus quality on lexical processing during sentence reading for middle-aged and older adult readers are lacking, and the results of previous work for younger adults is mixed. One possibility is that the manipulation of the stimulus quality for a single word in the text may serve to “highlight” this word, and may trigger processes that are not typical of normal reading (White & Staub, 2012). Therefore, in the present study, the stimulus quality of entire sentences was manipulated.

## Experiment 1

The present research expands on the previous work in three key ways. First, measures of eye movements provide more detailed insights into the time course of effects of text stimulus quality for older compared with young adults. Second, the research also examines effects of stimulus quality for middle-aged readers. Third, the effect of stimulus quality on lexical processing is examined by including a manipulation of word frequency. In line with previous work, it was predicted that reading times would be longer for sentences presented in low compared with high contrast for both young and older readers. If older readers are especially vulnerable to reductions in stimulus quality, reading faint text will disrupt eye movement behavior to a greater extent than for young adults. Middle-aged readers may also show greater vulnerability to reduced stimulus quality but to a lesser extent than older adults. Experiment 1 also included a manipulation of word frequency for a critical word within each sentence. If reduced text contrast increases the difficulty of lexical processing, there should be interactions between word frequency and stimulus quality, with larger effects of word frequency for faint text (in line with Sheridan & Reingold, 2013). It could be that these effects are exacerbated for middle-aged and older readers as a result of visual declines. Alternatively, it could be that the manipulation of stimulus quality for the entire sentence results in a different pattern of effects to those found in studies manipulating only a critical word, as shown by Liu et al. (2015) and Jainta et al. (2017).

### Method

#### Participants

Sixteen young, 16 middle-aged, and 16 older adults were recruited from Leicester, United Kingdom, and the surrounding community. All were native English speakers with no history of dyslexia or serious eye diseases. All three age groups had a similar educational background, reported engaging in regular reading, and had normal or corrected-to-normal vision, achieving at least 20/32 high-contrast acuity (corrected) at the viewing distance (assessed using ETDRS chart; Ferris & Bailey, 1996). A summary of these characteristics is shown in Table 1. In addition to the participants included in the analyses, six older adults were excluded because of an inability to read the low-contrast text.[Table-anchor tbl1]

#### Materials and design

**Figure 1 fig1:**
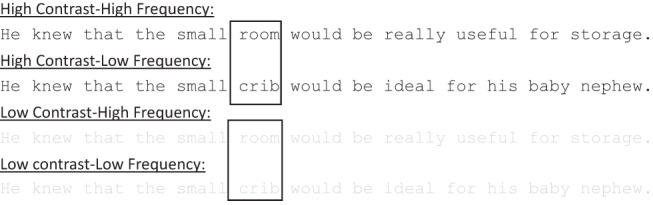
Experiment 1. An example sentence in each condition. A box highlights the high- or low-frequency critical word. This box was not present during the experiment.

The experiment was a 3 (age group: young, middle, older) × 2 (text contrast: normal text, faint text) mixed design, with an additional × 2 factor of word frequency (high, low) for the critical word analyses. Stimuli consisted of 120 sentences (from White, Drieghe, Liversedge, & Staub, 2018) including a high- or low-frequency four- to six-letter-long critical word (examples in Figure 1). A Latin square design ensured that each participant saw each critical word only once, with an equal number of sentences in each condition. Twenty-five percent of questions were followed by a comprehension question.[Fig-anchor fig1]

The luminance of the white background remained the same across all conditions (RGB 255 255 255; 54.25 cd/m^2^). The critical words were presented either in high contrast as black text (RGB 0 0 0; 0.53 cd/m^2^) or in low contrast as light gray text (RGB 217 217 217; 37.66 cd/m^2^).^1^

#### Apparatus and procedure

This study received ethical approval from The University of Leicester Ethics committee (Psychology). An SR Research tower mounted EyeLink 1000 eye tracker was used to record gaze location every millisecond. Viewing was binocular, but only the right eye was tracked. Stimuli were displayed in Courier New font on a 20-in. monitor with a screen resolution of 1024 × 768 and a display screen refresh rate of 120 Hz. At the 80-cm viewing distance, there were approximately 3.3 characters per degree of visual angle.

Participants were tested individually. Before commencing the experiment, the participant’s ability to read the low-contrast text was confirmed. The participant was then seated at the eye tracker, and a chin and forehead rest were used to minimize head movements. Prior to the presentation of the first sentence, a 3-point (left, center, and right) horizontal calibration and validation procedure was completed, with calibration checks between each trial. Calibrations were repeated as necessary. At the beginning of each trial, a fixation cross was presented in the same position as the beginning letter of each sentence. The participants were required to fixate this cross, which triggered the presentation of the text. Participants pressed a button on a response box once they had finished reading the sentence. The sentence then disappeared and was replaced on 25% of trials by a comprehension question that required a “yes” or “no” response, which participants gave using the response box. The experiment lasted approximately 30 min for each participant.

#### Analyses

Following standard procedures, fixations shorter than 80 ms or longer than 1,200 ms were discarded. This accounted for 1.8% of fixations. The data were analyzed using linear mixed effects models (LMMs; Baayen, Davidson, & Bates, 2008). LMMs were conducted using R (Version 3.2.3; R Core Team, 2015) and the lme4 package (Version 1.1–12; Bates, Mächler, & Bolker, 2011). Following current practice, a maximal random effects structure was used for continuous measures (following Barr, Levy, Scheepers, & Tily, 2013). Participants and stimuli were always specified as crossed-random effects. Generalized linear mixed models were conducted for dichotomous variables. For sentence-level measures, effects of age are first examined for only the normal (high contrast) condition (the results for these models are detailed in the text). For all of the conditions in the sentence-level analyses, age group, text contrast, and their interaction were entered as fixed effects, with frequency additionally included as a fixed effect in the critical word analyses.^2^ Results from these LMM models are reported in tables. Interaction effects were explored further with contrasts between pairs of variables. Contrasts were specified to compare the high- and low-contrast conditions for each age group, and when appropriate, to compare the high- and low-word-frequency conditions for each level of text contrast and age group. Contrasts to examine effects of age group (young vs. middle-aged; middle-aged vs. older), text contrast (normal text vs. faint text), and word frequency (high vs. low) were defined using sliding contrasts in the MASS package (Venables & Ripley, 2002; sliding contrasts were employed both for main effects and to examine interactions). We also examined effects of age using a young versus older contrast; however, the pattern of results was the same as for the middle-aged versus older contrast, and so, for brevity, we do not report these. For all analyses, *t* and *z* values >1.96 were considered significant.

Several standard sentence-level measures are reported: sentence reading time, average fixation duration, progressive saccade length (the length, in characters, of forward eye movements, both within and across words), number of first-pass skips (the number of words that do not receive a first-pass fixation), and number of regressions (number of backward eye movements, both within and across words). First-pass reading times (the sum of fixations that occurred the first time a word was encountered) and rereading times (the sum of rereading fixations) are also reported. Critical word measures are also reported: first-fixation duration^3^ (the duration of the first-fixation on a word during first-pass reading), gaze duration (the summed duration of all fixations on a critical word before a saccade is made out of the word), total reading time (the sum of all fixations on a word), and skipping probability (the probability of the critical word not receiving a first-pass fixation). All participants achieved a high level of comprehension accuracy in the experiment (Min = 85%).

### Results

#### Sentence-level analyses

Means and standard errors for sentence-level eye movement measures are shown in Table 2. Effects of age are first examined only for the normal high-contrast text condition. Compared with middle-aged adults, older adults produced longer sentence reading times (β = 475.58, *SE* = 240.46, *t* = 1.98), made more regressions (β = 0.65, *SE* = 0.10, *t* = 2.45), and produced longer rereading times (β = 509.20, *SE* = 167.64, *t* = 3.04), whereas the performance of middle-aged adults and young adults did not differ significantly (in all cases *t* < 1.20). These results reflect adult age differences in reading for those aged 65 and older compared with younger readers, in line with previous research (e.g., Rayner et al., 2006, 2009). Differences in average fixation duration, progressive saccade length, first-pass reading time, and number of first-pass skips did not reach significance (*t*s < 1.60). In contrast, previous studies have reported higher skipping rates for older compared with younger adults (e.g., Rayner et al., 2006). The intermingling of normal and faint sentences may have led older readers to adopt a more cautious reading strategy, reducing skipping rates for both normal and faint text conditions (see General Discussion). What seems especially important about the present results, however, is that under normal reading conditions, the eye movement behavior of young and middle-aged readers was very similar.[Table-anchor tbl2]

The results of the LMM for effects of age (young vs. middle-aged; middle-aged vs. older) and text contrast are summarized in Table 3. For progressive saccade length, there was an effect of stimulus quality (saccades were shorter for faint compared with normally presented text), but there were no effects of age or any interactions. For all of the other measures, there were no significant interactions between age and stimulus quality for young versus middle-aged adults; however, there were significant interactions between age and stimulus quality for middle-aged versus older adults. Crucially, stimulus quality modulated reading behavior for all three groups. Contrasts comparing the normal and faint conditions were undertaken for each age group. For middle-aged and older adults, there were longer sentence reading times, longer average fixation durations, fewer skips, more regressions, and longer first-pass and rereading times for faint compared with normally presented text (*t*s > 2). Similarly, for younger adults, there were significant effects of stimulus quality for sentence reading times, average fixation durations, first-pass reading time, and number of skips (*t*s > 2.9). However, for younger adults, there was no effect of stimulus quality for rereading times (β = 12.93, *SE* = 28.69, *t* = 0.45) or number of regressions (β = 0.14, *SE* = 0.08, *t* = 1.81). Importantly, the interactions between middle-aged versus older adults and stimulus quality reflect much larger effects of stimulus quality for the older adults compared with the other age groups. These results indicate that stimulus quality is particularly important for the reading of older adults, but that the vulnerability to reduced contrast is not yet present in middle-age.^4^

#### Critical word analyses

**Figure 2 fig2:**
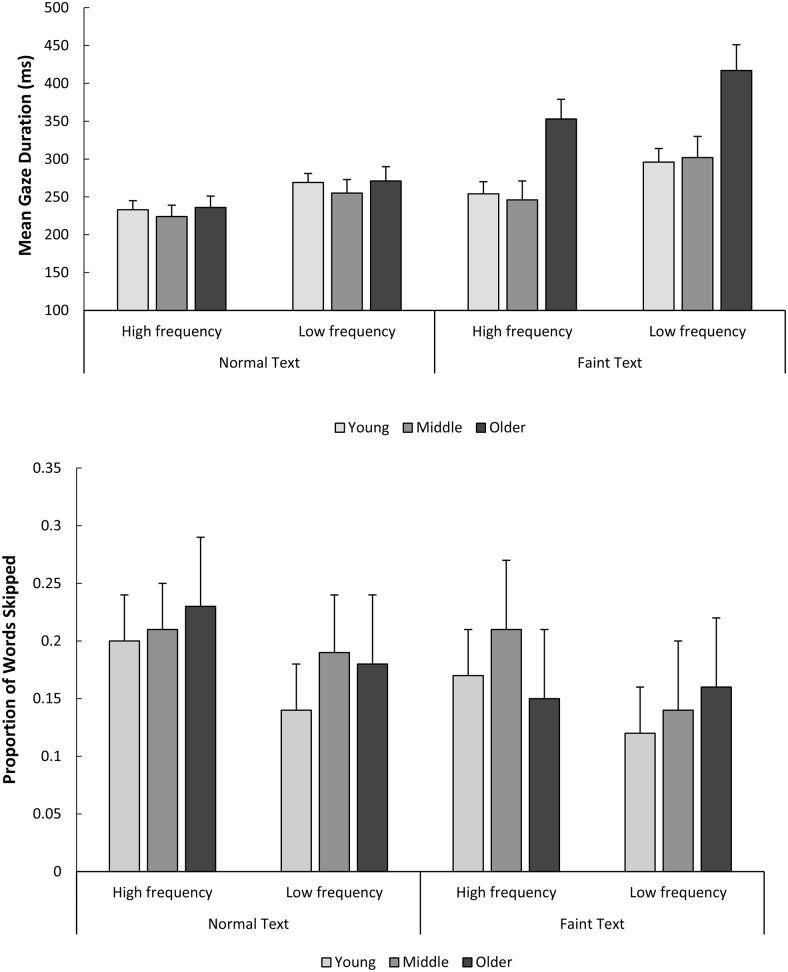
Experiment 1. Mean gaze durations (Panel A) and word skipping (Panel B) for the critical words. Error bars represent one standard error.

Means and standard errors for critical word analyses are shown in Table 4. The results of the LMM for effects of age (young vs. middle-aged; middle-aged vs. older), text contrast, and word frequency are summarized in Table 5. Follow-up contrasts to explore interactions are reported in the text. Results for gaze duration and word skipping are shown in Figure 2. For first-fixation duration, gaze duration, and total reading time, there were no three-way interactions. However, in line with the results for sentence-level measures, there were significant two-way interactions between age and stimulus quality for middle versus older, but not for young versus middle-aged, adults. Follow-up contrasts showed significant effects of stimulus quality for all age groups, with longer first-fixation durations, gaze durations, and total times for faint compared with normally presented words (*t*s > 2.9). In line with the sentence-level measures, effects of stimulus quality were larger for the older adults compared with both the younger and middle-aged adults. For first-fixation and gaze duration, there were no significant two-way interactions between age group and word frequency, but there was a significant interaction between word frequency and age group for total reading times. In line with the pattern shown in some previous studies, word frequency effects were larger for older adults compared with the other age groups.[Table-anchor tbl4][Table-anchor tbl5][Fig-anchor fig2]

For all three reading time measures, there were significant two-way interactions between stimulus quality and word frequency. All three measures were significantly longer for low-compared with high-frequency words for both faint text and normally presented text (*t*s > 5.20). However, in line with the results of Sheridan and Reingold (2013), the effects were larger for faint text than for normally presented text. Notably, despite interactions between stimulus quality and word frequency, these two-way interactions did not further interact with age group (in all cases, *t* < 1.10). Importantly, these results indicate that although compared with other age groups, the reading of older adults was more disrupted by poor stimulus quality, this was not related to particular difficulties with lexical processing.

In line with the reading time results, word skipping for young versus middle-aged adults produced no interactions but clear effects of both word frequency and stimulus quality. That is, in line with previous work, skipping rates were higher for high-compared with low-frequency words, and for normally presented compared with faint words. However, for the older adults, in contrast to the reading time measures, word skipping showed a three-way interaction between age (middle-aged vs. older), stimulus quality, and word frequency (see Figure 2, Panel B). For normally presented text, young and older adults showed significant effects of word frequency so that high-frequency words were more likely to be skipped than low-frequency words (*t*s > 2.00). Middle-aged adults showed the same numerical trend, though this did not reach significance (β = 0.09, *SE* = 0.07, *t* = 1.76). Crucially, for faint text, although word frequency modulated skipping rates for both young and middle-aged readers (*t*s > 2.00), no significant effects of word frequency were observed for older readers for faint text (β = 0.001, *SE* = 0.02, *t* = 0.26). This interaction may reflect an inability of older adults to lexically process low-contrast parafoveal text, suggesting that older adults experience particular difficulty processing faint text in the parafovea. This is consistent with the Age Group × Text Contrast interaction in the sentence-level word-skipping analyses.

### Discussion

Experiment 1 showed clear adult age differences in reading for participants aged 65+. These effects are consistent with those of previous studies (e.g., Rayner et al., 2006). Older adults produced significantly longer sentence reading times, and more regressions than both young and middle-aged adults. These differences in reading performance suggest that the older adults were experiencing greater reading difficulty. The middle-aged group produced broadly similar eye movement behavior to the young adults, indicating that reading processes and eye movement control are likely similar for middle-aged and young adults.

Stimulus quality affected various eye movement measures, for both first-pass and rereading. These effects are in line with previous studies with young adults that examined effects of reduced contrast for entire sentences (Hohenstein & Kliegl, 2014; Jainta et al., 2017; Liu et al., 2015; White & Staub, 2012). Also in line with previous research, words in the faint text condition were less likely to be skipped than normally presented words (Drieghe, 2008; Hohenstein & Kliegl, 2014; Sheridan & Reingold, 2013). Importantly, older readers demonstrated greater disruption for faint text compared with both young and middle-aged adult readers. This was found across various eye movement measures and builds on previous research demonstrating greater increases in reading time (Mitzner & Rogers, 2006). Together, this work suggests that older adults are particularly vulnerable to the effects of low-contrast text. Crucially, these additional difficulties in reading low-contrast text were not seen for middle-aged participants. This suggests that despite the onset of neural and optical changes occurring in middle age (Owsley, 2011; Schefrin et al., 1999), reading difficulties associated with advanced age do not affect reading behavior for middle-aged adults.

A main effect of word frequency was found across all measures. Frequency also interacted with contrast for the reading time measures. This interaction emerged early, from the duration of the first fixation, and was present throughout the reading process. This finding provides support for the notion that stimulus quality has an early influence on lexical processing. Therefore, this suggests that when text contrast is reduced, word identification is made more difficult. This is in line with results from single-word recognition studies, which have found interactive effects of stimulus quality and word frequency in semantic categorization and pronunciation tasks (O’Malley et al., 2007; Yap & Balota, 2007). It is also consistent with the results of Sheridan and Reingold (2013), who used faint critical words embedded within sentences to examine effects of stimulus quality and word frequency in natural reading. However, these findings differ from the additive effects shown in lexical decision studies (e.g., Becker & Killion, 1977) and also for entire-sentence manipulations of stimulus quality (Jainta et al., 2017; Liu et al., 2015). The possible role of orthography in mediating the effects during sentence reading is considered further in the General Discussion. Crucially, the influence of reduced contrast on word identification on reading times was similar for the three age groups. These findings provide an important indication that, although overall, compared with other age groups, the reading of older adults was more disrupted by reduced text contrast, this was not because of additional difficulties associated with lexical processing. Experiment 2 builds on this work by examining the effect of text contrast for parafoveal text.

## Experiment 2

Experiment 1, and most previous studies examining effects of stimulus quality on reading behavior, manipulated the stimulus quality for words prior to, during, and after fixation (that is, both in the fovea and parafovea). However, few studies have specifically examined how text contrast modulates processing of fixated words (Glaholt et al., 2014) and parafoveal words (Hohenstein & Kliegl, 2014; see also: Wang & Inhoff, 2010).^5^ Visual contrast is known to affect recognition of stimuli outside of central vision (Strasburger, Rentschler, & Jüttner, 2011), and parafoveal processing is known to be a crucial component of skilled reading (see Schotter, Angele, & Rayner, 2012). Reducing text contrast may be particularly disruptive to parafoveal processing of upcoming words for older readers. Many of the changes in visual abilities that occur during the normal aging process are greatest outside of central vision, including age-related declines in visual acuity and contrast sensitivity (Collins, Brown, & Bowman, 1989; Crassini et al., 1988; Gillespie-Gallery, Konstantakopoulou, Harlow, & Barbur, 2013). Older age is additionally associated with greater effects of visual crowding, characterized by a difficulty in identifying visual objects when in close proximity to other, similar visual objects, such as letters, and these effects are particularly pronounced in parafoveal vision (McCarley, Yamani, Kramer, & Mounts, 2012; Scialfa, Cordazzo, Bubric, & Lyon, 2013). Therefore, Experiment 2 aimed to examine how text contrast in the parafovea modulates reading behavior and whether young and older readers are differentially affected by a reduction in text contrast in the parafovea. As little difference was observed between young and middle-aged adults in Experiment 1, Experiment 2 focused only on young and older adult readers.

**Figure 3 fig3:**
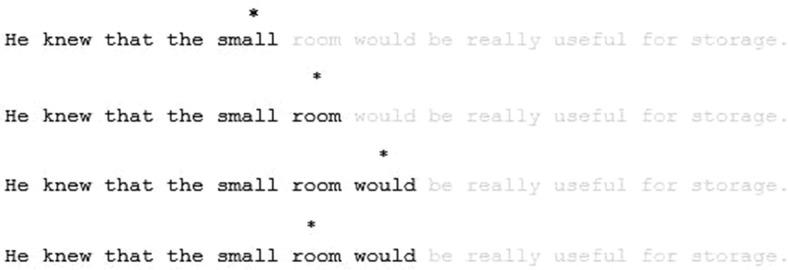
Experiment 2. A demonstration of the gaze-contingent change in the low-contrast upcoming text condition. The asterisk represents the point of fixation. Once an invisible boundary is crossed before each word, the word changes to high contrast; this word remains high contrast if a regression is made.

Experiment 2 employed a variation of the gaze-contingent text change technique (McConkie & Rayner, 1975) to present upcoming words in the parafovea in low contrast (see Figure 3). The fixated word and all previously encountered words were presented normally in high contrast, whereas upcoming text was presented in low contrast and thus appeared faint. Note that in contrast to studies that have examined parafoveal preview effects (Vasilev & Angele, 2017), the orthography of the parafoveal previews was always correct. The current manipulation bears some similarity to other gaze-contingent methods (Marx, Hawelka, Schuster, & Hutzler, 2015; Marx, Hutzler, Schuster, & Hawelka, 2016; Rayner, Yang, Schuett, & Slattery, 2013). However, a crucial difference is that the current manipulation was employed only for first-pass reading, so that all words to the left of fixation were presented normally and remained at high contrast during rereading.[Fig-anchor fig3]

Given the changes in visual abilities across the adult life span, young readers are likely to be better able to linguistically process low-contrast parafoveal text and better able to use low-contrast parafoveal text for saccade programming. Therefore, we anticipate that the reading of older readers will be disrupted more than that of younger readers by faint upcoming text to the right of fixation. As upcoming text was always orthographically correct, the pattern of predicted age differences is different than that for studies that assess preview benefit by comparing correct and incorrect orthographic previews (e.g., Rayner, Castelhano, & Yang, 2010). In preview benefit studies, neither age group can extract useful information from an incorrect preview, and young adults can benefit more from the correct preview; hence, the preview effect is larger (more benefit) for young adults. In contrast, in the present study, it is predicted that young adult readers extract more useful information than older adults from low-contrast upcoming text; hence, the effect of this manipulation is predicted to be larger (and thus more detrimental) for older adults. Note that for consistency, a word frequency manipulation is included in Experiment 2. However, as fixated words are always presented at high contrast in Experiment 2, it was not anticipated that stimulus quality would modulate the size of the word frequency effect.

### Method

#### Participants

Sixteen young adults and 16 older adults were recruited from the University of Leicester and the surrounding community. None took part in Experiment 1. Criteria for participating were the same as in Experiment 1 and participants’ visual abilities were assessed using the same tests, as summarized in Table 1. Three older adults were excluded because of an inability to read foveally presented low-contrast text.

#### Materials and design

The design of the experiment was a 2 (age: young, older) × 2 (upcoming text: normal, faint) mixed design with an additional × 2 factor of word frequency (high, low) for the critical word analyses. The same materials and Latin square design were employed as in Experiment 1. In the faint condition upcoming text was presented at low contrast during first-pass reading (see Figure 3). A boundary was placed between every word, so that each word was always presented at high contrast at the point of fixation, and upcoming text was presented at low contrast. This manipulation was employed only during first-pass reading. Therefore, once each boundary was crossed the word remained at high contrast (including during rereading).

#### Apparatus and procedure

Experiment 2 used the same apparatus and general procedure as Experiment 1.

#### Analyses

Experiment 2 used the same measures and data analysis procedures as Experiment 1. For Experiment 2, 2% of fixations were shorter than 80ms or longer than 1,200ms, and so these fixations were discarded. All participants achieved a high level of comprehension accuracy in the experiment (at least 85%).

### Results

#### Sentence-level analyses

Means and standard errors for sentence-level analyses are shown in Table 6. Adult age differences in line with Experiment 1 were found for normally presented text. Older adults produced longer reading (β = 442.10, *SE* = 213.50, *t* = 2.07) and fixation times (β = 19.04, *SE* = 8.83, *t* = 2.16), made longer progressive saccades (β = 0.51, *SE* = 0.17, *t* = 3.15) and more regressions (β = 1.10, *SE* = 0.90, *t* = 2.30), and spent more time rereading (β = 74.17, *SE* = 27.64, *t* = 2.68) than young adults. No significant age effects were found for number of first-pass skips or first-pass reading times (all *ts* < 1.20). Accordingly, although the older adults read more slowly than the young adults, differences in word skipping were not observed across the two age groups. As in Experiment 1, these findings are broadly in line with previous research (e.g., Kliegl et al., 2004; Rayner et al., 2006, 2009) but, similar to Experiment 1, did not show significant age differences in word-skipping (see General Discussion).[Table-anchor tbl6]

The results of the LMM for effects of age (young vs. older) and text contrast are summarized in Table 7. There were significant effects of age group for measures sensitive to rereading behavior (number of regressions and rereading time). For these measures, there were no effects of upcoming text contrast and no interactions. Therefore, when stimulus quality is manipulated both foveally and parafoveally (Experiment 1), text contrast modulated rereading behavior, but when stimulus quality was only manipulated for upcoming words to the right of fixation (Experiment 2), there was no effect of text contrast on rereading behavior.[Table-anchor tbl7]

In contrast to measures sensitive to rereading, all other sentence-level measures produced significant interactions between age and upcoming text stimulus quality, such that effects of stimulus quality were larger for older than younger adults. Both groups produced significantly longer sentence reading times, fixation durations, and first-pass reading times; significantly fewer first-pass skips; and significantly shorter progressive saccade lengths in the faint compared with the normal upcoming text condition (*t*s > 2.4), but these effects were larger for the older adults. Overall, the sentence-level results suggest that both age groups benefit from the availability of high-visual-quality text in the parafovea. However, in line with predictions, older adults appear to have greater difficulty in processing parafoveal text when it is presented in low contrast.

#### Critical word analyses

Means and standard errors for the critical word analyses are shown in Table 8. The results of the LMM for effects of age (young vs. middle-aged; middle-aged vs. older), text contrast, and word frequency are summarized in Table 9, follow-up contrasts are reported in the text. There were significant effects of age and upcoming text contrast for all three reading time measures for the critical word, and significant interactions between these factors. For first-fixation duration and gaze duration, there were significant effects of upcoming text contrast for older (first-fixation duration, β = 21.04, *SE* = 4.05, *t* = 5.20; gaze duration, β = 27.37, *SE* = 4.12, *t* = 6.64) but not younger (first-fixation duration, β = 2.99, *SE* = 4.08, *t* = 0.73; gaze duration, β = 5.75, *SE* = 4.02, *t* = 1.43) adults. Both groups produced significantly longer total times for faint compared with normal upcoming text (*t*s > 1.96). The interaction reflects a larger effect for older compared with young adults. In line with the sentence-level measures, these results indicate that both age groups benefit from high-contrast parafoveal text but older adults have particular difficulty processing low-contrast parafoveal text.[Table-anchor tbl8][Table-anchor tbl9]

In line with Experiment 1 and previous studies, there were significantly longer reading times for low-compared with high-frequency words. There were no significant interactions between word frequency and age (though first-fixation durations and total times did show a numerically larger frequency effect for older readers). In contrast to Experiment 1, the results for reading times on the critical word showed no interaction between word frequency and upcoming text contrast, indicating that the interactive pattern in Experiment 1 reflects foveal processing of the critical word rather than an effect of the stimulus quality of parafoveal text. There were also no three-way interactions for any reading time measures. Therefore, having the foveal word presented intact appears to support normal lexical processing for both age groups even when the contrast of text in the parafovea is low.

For word skipping, there was a significant effect of word frequency and a significant interaction between age group and upcoming text contrast. The interaction was such that when collapsed across the word frequency conditions, young adults, but not older adults, appeared to have higher skipping rates when upcoming text was presented at low contrast. However, this pattern must be interpreted with caution, as it contrasts with the pattern of word-skipping effects in the sentence-level analyses for this experiment, and also the word-skipping effects observed in Experiment 1. Recall that in Experiment 1 there was a significant effect of stimulus quality on word skipping that was qualified by a three-way interaction, such that young readers showed effects of word frequency on word skipping regardless of stimulus quality, whereas word frequency only modulated word skipping for normally presented text for older readers. In contrast, for Experiment 2, there was no significant three-way interaction. However, for both groups, word frequency effects were numerically smaller for low-compared with high-contrast upcoming text, though the interaction between word frequency and stimulus quality did not reach significance (*t* = 1.78).

### Discussion

In line with previous studies and the results of Experiment 1, adult age differences were found, with older adults showing longer sentence reading times and more regressions than young adults. This further demonstrates that older adults experience greater reading difficulty (e.g., Rayner et al., 2006). Effects of upcoming text contrast were shown for both age groups in sentence-level analyses, suggesting that both groups benefitted from the availability of high-contrast text in the parafovea. These results build on previous findings (Hohenstein & Kliegl, 2014), suggesting that the contrast of upcoming text contributes to reading difficulties for text presented entirely at low contrast (both foveally and parafoveally; e.g., Reingold & Rayner, 2006; White & Staub, 2012). The effects of upcoming text contrast on reading times are likely a result of parafoveal preprocessing (that is, the influence of parafoveal text on word skipping probabilities and subsequent reading times). However, note that effects could also arise as a result of parafoveal-on-foveal effects (that is, the influence of parafoveal text characteristics on reading behavior for preceding words).^6^ Crucially, older adults showed greater increases in reading time than young adults when text presented in the parafovea was faint. This suggests that older adults experience greater difficulty than young adults in processing parafoveal information in the absence of a high-contrast preview. For the young adults, superior visual abilities may enable them to extract more information from low-contrast previews compared with older adults.

Interestingly, effects of word frequency and upcoming text contrast produced additive effects for critical word reading times. It appears that when the foveal word is presented normally, lexical processing of fixated words is not modulated by upcoming text contrast. The parafoveal preview is likely to be especially important in the very early stages of word recognition, and it may be that the processes underlying the interactive pattern in Experiment 1 occur at a later stage—that is, primarily during the foveal, rather than parafoveal, processing of words. The additive effects of word frequency and upcoming text contrast for both young and older adults are especially striking given that previous studies show smaller or delayed effects of word frequency when accurate previews of the critical word are denied (Inhoff & Rayner, 1986; Rayner et al., 2006; Reingold, Reichle, Glaholt, & Sheridan, 2012). In contrast, in the present study, although low-contrast previews resulted in longer reading times, there was no apparent detriment to lexical processing. However future studies may employ distributional analyses to further examine whether the stimulus quality of upcoming text modulates the time course of lexical influences on fixation durations (see Reingold et al., 2012), with potentially important implications for theoretical models of the underlying mechanisms (Sheridan & Reichle, 2016).

## General Discussion

The present study yielded three key novel findings:

1 Adult age differences in reading normal (high-contrast) text: In addition to effects of older age in line with previous studies, Experiment 1 further demonstrated that the reading behavior of middle-aged readers is comparable to that of younger adults.
2 Effects of text contrast: Eye movement behavior of older readers is substantially more disrupted by faint (low-contrast) text presentation compared with younger readers, both for text presented entirely in low contrast (Experiment 1) and for parafoveal modulations of text contrast (Experiment 2).
3 Stimulus quality and lexical processing: In line with Sheridan and Reingold (2013), Experiment 1 demonstrated an interactive effect of stimulus quality and word frequency, and, crucially, the same pattern is shown to hold for older readers.

Overall, although older adults show substantially more disruption from low-contrast text presentations, this difficulty appears to impact largely at a visual level of text processing, with no particular disruption at the lexical level. Each of these key novel findings is discussed further in the three sections that follow.

### Adult Age Differences in Reading Normal (High-Contrast) Text

In line with previous studies, the experiments reported here clearly show that older adults (aged 65+ years) experience greater reading difficulty than young adults (aged 18–24 years). Even when reading normally presented text, the older adults read more slowly and made longer fixations and more regressions than young adults. The broad pattern of this age-related reading difficulty is similar to that reported previously (e.g., Kliegl et al., 2004; McGowan et al., 2014, 2015; Paterson et al., 2013a; Rayner et al., 2006, 2009; Stine-Morrow et al., 2010). The results of Experiment 1 also provide some indication that older adults produce larger word frequency effects than young adults by making disproportionately longer fixations on infrequent words (Kliegl et al., 2004; McGowan et al., 2015; Rayner et al., 2006, 2013), although this pattern did not reach significance in Experiment 2. Unlike many previous experiments (Kliegl et al., 2004; McGowan et al., 2014, 2015; Rayner et al., 2006; Warrington et al., 2018; but see Choi et al., 2017; Whitford & Titone, 2016, 2017), we did not observe increased word skipping for older compared with younger adults. Higher skipping rates for older adults have been attributed to a change in reading strategy to compensate for slower processing of words in older age (e.g., Rayner et al., 2006) or less flexible saccade programming (see Risse & Kliegl, 2011; Wotschack & Kliegl, 2013). However, this characterization of age changes in reading behavior is a matter of current debate (e.g., Choi et al., 2017), and, as we argue in the next section, the tendency for older adults to skip words more often may be lessened in more difficult reading conditions (see Wotschack & Kliegl, 2013).

Experiment 1 also compared the reading of middle-aged readers to young and older readers. Previous studies hinted at age differences in reading for those in middle age (Calabrèse et al., 2016; Soederberg Miller, 2009; see also Teramoto et al., 2012). However, the maximum age of participants in these studies (59 years) was higher than for the middle-aged participants included here (51 years). The results of Experiment 1 provide a promising initial indication that age-related declines in reading performance are not yet present for middle-aged readers (at least within the range of 41 to 51 years), despite the onset of neural and optical changes beginning around 40 years of age (Owsley, 2011; Schefrin et al., 1999).

### Effects of Text Contrast

In line with Mitzner and Rogers (2006), the present study shows larger effects of text contrast on reading times for older adults. Building on this work, Experiment 1 is one of the first to employ eye movement measures to examine this difference in detail. The results showed that reducing the contrast of all words within a sentence disrupts normal eye movement behavior (both first-pass and rereading behavior) more for older—than for young or middle-aged readers—who showed similar performance to young adult readers. Experiment 2 additionally found that reducing the contrast of all upcoming words within a sentence disrupts normal eye movement behavior more for older than for young readers. These results indicate that older adults are less able to make use of low-contrast text in the parafovea. The indication, therefore, is that the reading performance of older adults is especially vulnerable to reductions in stimulus quality both in the fovea and in the parafovea.^7^ This pattern was found consistently across a number of reading times measures. In addition, although the pattern of results for word skipping on the target word was more complex, there are some key similarities between the two experiments. In both, older adults reduced their skipping in the faint text condition more than young adults. This was seen in sentence-level analyses in Experiment 1 and in both sentence-level and critical word analyses in Experiment 2. Together, the results are consistent with previous research showing that older adults skip less in more difficult reading conditions (see Wotschack & Kliegl, 2013). Given the intermingling of the normal and faint sentences, older adults’ expectation that they may encounter faint text may have resulted in a more cautious reading strategy throughout the experiment, for both the normal and the faint sentences. That is, the increased difficulty associated with the low-contrast text presentations may have prompted older adults in the present experiments to adopt a more careful reading strategy than in previous studies (e.g., Rayner et al., 2006).

The numerous visual declines that occur in older age are likely to be a key component in this differential response to reduced stimulus quality. In particular, older age results in a gradual loss of sensitivity to fine visual detail so that higher contrast is often required (Owsley, 2011). This may be an important component of the reading difficulty that older adults experience. Accounting for effects of stimulus quality may therefore be crucial in the development of models of eye movement control (e.g., Engbert, Nuthmann, Richter, & Kliegl, 2005; Reichle, Rayner, & Pollatsek, 2003) that account for reading behavior across the life span.

### Stimulus Quality and Lexical Processing

A further concern was to establish if a reduction in stimulus quality affects only the early encoding of visual features or also the subsequent lexical processing of words. The answer has implications for understanding word recognition processes and eye movement control mechanisms. The results of recent studies on effects of text contrast and word frequency for young adults are inconsistent. The results in the present study are consistent with previous studies for which the stimulus quality of only a single critical word in the sentence was manipulated. Such studies have shown an interactive pattern of results, with larger effects of word frequency for words presented at low contrast, consistent with an influence of stimulus quality on lexical processing (Liu et al., 2015; Sheridan & Reingold, 2013). However, experiments that manipulated the stimulus quality of the entire sentence have shown additive effects of the two variables (Jainta et al., 2017; Liu et al., 2015), in line with an effect of stimulus quality at visual stages of processing (e.g., feature extraction) but not lexical stages. These additive results contrast with the interactive pattern shown here. Liu et al.’s study employed Chinese text and also a very low-contrast stimulus quality manipulation, and either or both of these factors could have contributed to a different pattern of results. However, differences in results between the present study and those of Jainta et al. point to the possibility that even relatively subtle differences in orthography might modulate effects of stimulus quality.

Jainta et al.’s (2017) study employed German text, for which critical words could be capitalized (as is standard for German nouns). Capitalization has been shown to influence how words are processed, perhaps because of the visual salience of the initial letter (Hohenstein & Kliegl, 2013; Rayner & Schotter, 2014). Interestingly, in Experiment 1 text contrast influenced all three reading time measures on the critical word. In contrast in Jainta et al.’s study, which employed a similar text contrast manipulation, text contrast influenced first-fixation durations but not gaze durations or total time on the critical words. It could be that the visually salient capitalization cues in Jainta et al.’s study facilitated orthographic processing of these words, mitigating the effects of the low-contrast presentation format. The interaction between stimulus quality and word frequency shown here and in Sheridan and Reingold’s (2013) study may hold in the absence of visually salient orthographic cues.

Crucially, effects of stimulus quality on lexical processing in the present study showed the same interactive pattern across the three adult age groups. Importantly, these results indicate that although when compared with other age groups, older adults’ reading was more disrupted by reducing text contrast, this did not result from additional difficulties in word recognition. Notably, although Experiment 1 produced interactive effects of contrast and word frequency, there were additive effects of word frequency and upcoming text contrast in Experiment 2, suggesting that when the foveal word is presented normally and parafoveal information is presented with contrast reduced, lexical processing is not affected. Rayner et al. (2006) employed the E-Z Reader model to produce simulations of older adults’ reading behavior by modulating just a few of the key parameters within the model. These simulations included changes to parameter ε, which modulates the effect of visual acuity limitations on the rate of lexical processing. However, the present study suggests that stimulus quality affects older adults’ reading behavior independently of lexical processing. It therefore could be that other mechanisms also are crucial in accounting for changes in the effects of stimulus quality across the life span, such as the duration of the V stage in E-Z reader. “V” relates to preattentive visual processing. In this stage, low-spatial-frequency information enables programming of saccades to words and high-spatial-frequency information enables letter features to be processed. Stimulus quality may modulate the rate of preattentive visual processing within the V stage (see White & Staub, 2012). Accordingly, the time required for completion of preattentive visual processing may be longer for low-compared with high-contrast text, and this may especially be the case for older readers.

## Conclusion

In conclusion, the present experiments provide novel insights into the effects of text contrast on eye movements during reading across the life span. Older readers suffer more than young adults from reductions in text contrast. This increased difficulty is experienced both for text presented entirely at low contrast and when parafoveal text is presented at low contrast. However, although reducing the contrast of all words in a sentence was found to modulate lexical processing, this effect was similar for all age groups. Thus, the additional difficulty incurred by modulation of text contrast primarily affects older adults’ visual, rather than lexical, processing of text. Overall, the current study indicates that, in addition to other age-related changes, text contrast may be an important source of reading difficulty for older adults.

## Figures and Tables

**Table 1 tbl1:** Experiments 1 and 2: Summary of Mean Participant Characteristics (Range Shown in Parentheses)

Experiments 1 and 2	Age (years)	Formal education (years)	Regular reading (hr/week)	Screen distance acuity	Contrast sensitivity
Experiment 1					
Young adults	19.2 (18–22)	15 (13–18)	11.5 (2–25)	20/17 (20/14–20/25)	1.97 (1.90–2.10)
Middle-aged adults	46.4 (41–51)	14.2 (11–18)	13 (2–30)	20/20 (20/14–20/26)	1.95 (1.90–2.05)
Older adults	69 (65–79)	15.7 (12–22)	12.4 (4–28)	20/25 (20/16–20/32)	1.94 (1.90–1.95)
Experiment 2					
Young adults	20.1 (18–24)	14.5 (13–18)	12.1 (4–30)	20/18 (20/16–20/25)	2.00 (1.95–2.10)
Older adults	69 (65–74)	13.7 (11–20)	12.8 (5–25)	20/24 (20/18–20/32)	1.95 (1.95–2.00)

**Table 2 tbl2:** Experiment 1: Means and Standard Errors (in Parentheses) for Sentence-Level Measures

Experiment 1	Normal text	Faint text
Sentence reading time (ms)		
Young	2,508 (169)	2,699 (270)
Middle	2,569 (240)	2,952 (350)
Older	3,040 (240)	4,465 (350)
Average fixation duration (ms)		
Young	236 (7)	251 (10)
Middle	237 (10)	259 (13)
Older	251 (10)	310 (13)
Progressive saccade length (characters)		
Young	7.9 (.2)	7.8 (.2)
Middle	8.5 (.2)	8.2 (.2)
Older	8.3 (.2)	8.0 (.2)
Number of first-pass skips		
Middle	5.1 (.4)	4.7 (.4)
Older	5.1 (.4)	4.4 (.5)
Number of regressions		
Young	2.4 (.2)	2.2 (.2)
Middle	2.3 (.2)	2.5 (.3)
Older	3.0 (.3)	3.5 (.4)
First-pass reading time (ms)		
Young	1,867 (115)	2,052 (146)
Middle	1,872 (162)	2,132 (206)
Older	1,970 (163)	2,821 (207)
Rereading time (ms)		
Young	530 (113)	543 (171)
Middle	552 (113)	680 (159)
Older	837 (160)	1,357 (242)

**Table 3:** Linear Mixed Model Statistics for Sentence-Level Measures

Experiment 1	Sentence reading time (ms)	Average fixation duration (ms)	Progressive saccade length (characters)	Number of first-pass skips	Number of regressions	First-pass reading time (ms)	Rereading time (ms)
Age							
Young vs. Middle							
β	166.66	32.18	.19	.06	.16	73.64	157.96
*SE*	284.42	11.24	.20	.45	.39	174.72	178.27
*t*	.58	.44	.97	.13	.41	.42	.89
Middle vs. Older							
β	1,000.40	32.18	.11	.15	.73	363.77	680.77
*SE*	284.45	11.24	.20	.45	.39	174.72	178.00
*t*	3.52*	2.82*	.57	.33	1.94	2.08*	3.32*
Text contrast							
Normal vs. Faint							
β	668.38	31.70	.12	.39	.20	448.96	210.10
*SE*	84.58	3.29	.02	.33	.08	44.35	53.54
*t*	7.92*	9.42*	6.97*	12.02*	2.42*	10.12*	3.92*
Interactions							
Young vs. Middle × Contrast							
β	194.25	6.32	.03	.05	.30	127.84	68.03
*SE*	206.62	8.04	.04	.08	.20	108.18	129.92
*t*	.94	.79	.74	.64	1.37	1.18	.52
Middle vs. Older × Contrast							
β	1,045.49	38.93	.06	.36	.40	535.34	463.28
*SE*	206.79	8.05	.04	.08	.20	108.18	129.80
*t*	5.05*	4.84*	1.48	4.55*	1.98*	4.95*	3.60*
*Note.* Significant effects are indicated with an asterisk.							

**Table 4 tbl4:** Experiment 1: Means and Standard Errors (in Parentheses) for Critical Word Measures

Experiment 1	Normal text		Faint text	
	High frequency	Low frequency	High frequency	Low frequency
First-fixation duration (ms)				
Young	215 (7)	234 (8)	232 (12)	260 (14)
Middle	212 (7)	227 (10)	234 (12)	266 (20)
Older	220 (10)	244 (11)	311 (17)	345 (20)
Gaze duration (ms)				
Young	233 (12)	269 (16)	254 (18)	296 (26)
Middle	224 (15)	255 (18)	246 (25)	302 (28)
Older	236 (15)	271 (19)	353 (26)	417 (34)
Total reading time (ms)				
Young	278 (24)	312 (25)	284 (30)	342 (47)
Middle	263 (30)	298 (30)	299 (44)	361 (65)
Older	298 (28)	359 (29)	491 (42)	614 (65)
Proportion of words skipped				
Young	.20 (.04)	.14 (.04)	.17 (.04)	.12 (.04)
Middle	.21 (.04)	.19 (.05)	.21 (.06)	.14 (.06)
Older	.23 (.06)	.18 (.06)	.15 (.06)	.16 (.06)

**Table 5 tbl5:** Experiment 1: Linear Mixed Model Statistics for Critical Word Measures

Experiment 1	First-fixation duration (ms)	Gaze duration (ms)	Total reading time (ms)	Proportion of words skipped
Age				
Young vs. Middle				
β	2.13	4.88	.22	.04
*SE*	12.88	18.22	35.15	.05
*t*/*z*	.17	.27	.01	.74
Middle vs. Older				
β	49.96	61.96	143.43	.02
*SE*	12.88	18.24	35.15	.05
*t*/*z*	3.64*	3.40*	4.08*	.37
Frequency				
High vs. Low				
β	26.13	46.20	68.89	.05
*SE*	2.18	3.39	5.38	.01
*t*/*z*	11.99*	13.61*	12.80*	5.36*
Text contrast				
Normal vs. Faint				
β	.35	64.40	99.93	.03
*SE*	.08	7.46	14.55	.01
*t*/*z*	10.34*	8.63*	6.87*	2.74*
Age × Frequency				
Young vs. Middle × Frequency				
β	.47	4.23	3.30	.01
*SE*	5.37	8.32	13.27	.02
*t*/*z*	.09	.51	.25	.08
Middle vs. Older × Frequency				
β	5.21	13.82	57.99	.04
*SE*	5.34	8.36	13.21	.02
*t*/*z*	.98	1.65	4.39*	1.73
Age × Text Contrast				
Young vs. Middle × Contrast				
β	9.20	11.09	30.57	.01
*SE*	11.61	17.74	35.32	.02
*t*/*z*	.79	.63	.87	.55
Middle vs. Older × Contrast				
β	65.31	99.57	184.99	.04
*SE*	11.60	17.79	35.30	.02
*t*/*z*	5.63*	5.60*	5.24*	1.49
Text Contrast × Frequency				
Contrast × Frequency				
β	11.53	22.53	55.75	.01
*SE*	4.36	6.79	10.77	.02
*t*/*z*	2.64*	3.32*	5.18*	.07
Age × Text Contrast × Frequency				
Young vs. Middle × Contrast × Frequency				
β	6.69	15.17	3.60	.11
*SE*	10.75	16.64	26.55	.05
*t*/*z*	.62	.91	.14	1.44
Middle vs. Older × Contrast × Frequency				
β	5.95	12.81	40.99	.14
*SE*	10.69	16.75	26.44	.05
*t*/*z*	.56	.76	1.06	3.15*
*Note.* Significant effects are indicated with an asterisk.				

**Table 6 tbl6:** Experiment 2: Means and Standard Errors (in Parentheses) for Sentence-Level Measures

Experiment 2	Normal upcoming text	Faint upcoming text
Sentence reading time (ms)		
Young	2,344 (170)	2,481 (250)
Older	2,800 (280)	3,406 (350)
Average fixation duration (ms)		
Young	212 (6)	222 (6)
Older	232 (7)	252 (7)
Progressive saccade length (characters)		
Young	7.26 (.1)	7.03 (.1)
Older	8.94 (.1)	7.85 (.1)
Number of first-pass skips		
Young	4.5 (.3)	4.1 (.3)
Older	4.9 (.3)	4.0 (.3)
Number of regressions		
Young	2.1 (.2)	2.1 (.2)
Older	3.0 (.2)	3.1 (.2)
First-pass reading time (ms)		
Young	1,940 (106)	2,065 (109)
Older	2,067 (150)	2,606 (154)
Rereading time (ms)		
Young	468 (61)	468 (64)
Older	638 (86)	683 (91)

**Table 7 tbl7:** Experiment 2: Linear Mixed Model Statistics for Sentence-Level Measures

Experiment 2	Sentence reading time (ms)	Average fixation duration (ms)	Progressive saccade length (characters)	Number of first-pass skips	Number of regressions	First-pass reading time (ms)	Rereading time (ms)
Age							
β	669.99	25.56	.12	.20	.25	321.29	99.66
*SE*	208.29	8.63	.49	.20	.11	148.03	38.30
*t*	3.36*	2.96*	.25	.49	2.48*	2.17*	2.78*
Upcoming text contrast							
β	358.09	13.72	.76	.60	.01	337.52	2.36
*SE*	38.82	1.65	.09	.30	.04	29.48	11.35
*t*	9.22*	8.30*	8.65*	8.42*	.07	11.45*	.10
Age × Upcoming Text Contrast							
β	471.14	10.68	.86	.60	.04	418.59	21.94
*SE*	77.62	3.31	.17	.10	.08	58.97	22.20
*t*	6.07*	3.23*	4.90*	4.74*	.52	7.10*	1.13
*Note.* Significant effects are indicated with an asterisk.							

**Table 8 tbl8:** Experiment 2: Means and Standard Errors (in Parentheses) for Critical Word Measures

Experiment 2	Normal upcoming text		Faint upcoming text	
	High frequency	Low frequency	High frequency	Low frequency
First-fixation duration (ms)				
Young	201 (6)	217 (9)	205 (7)	219 (9)
Older	228 (9)	255 (12)	251 (11)	273 (13)
Gaze duration (ms)				
Young	212 (9)	252 (14)	222 (9)	248 (17)
Older	239 (12)	281 (20)	272 (13)	309 (24)
Total reading time (ms)				
Young	241 (14)	291 (23)	249 (17)	295 (27)
Older	301 (20)	366 (32)	332 (24)	401 (38)
Proportion of words skipped				
Young	.20 (.03)	.14 (.03)	.23 (.04)	.22 (.04)
Older	.23 (.05)	.17 (.05)	.18 (.05)	.17 (.04)

**Table 9 tbl9:** Experiment 2: Linear Mixed Model Statistics for Critical Word Measures

Experiment 2	First-fixation duration (ms)	Gaze duration (ms)	Total reading time (ms)	Proportion of words skipped
Age				
β	39.75	44.08	81.34	.14
*SE*	10.46	15.68	22.96	.33
*t*/*z*	3.80*	2.81*	3.54*	.43
Frequency				
β	19.82	36.68	58.30	.43
*SE*	2.37	3.18	5.48	.13
*t*/*z*	8.37*	11.55*	10.64*	3.30*
Upcoming text contrast				
β	12.49	16.91	19.00	.12
*SE*	2.37	3.18	5.48	.20
*t*/*z*	5.28*	5.33*	3.47*	.95
Age × Frequency				
β	8.47	6.52	17.68	.10
*SE*	4.73	6.35	10.96	.23
*t*/*z*	1.79	1.03	1.61	.48
Age × Contrast				
β	18.52	31.31	27.86	.67
*SE*	4.73	6.35	10.96	.23
*t*/*z*	3.91*	4.93*	2.54*	2.93*
Contrast × Frequency				
β	4.06	9.41	.62	.40
*SE*	4.73	6.35	10.96	.23
*t*/*z*	.86	1.48	.06	1.78
Age × Contrast × Frequency				
β	4.74	9.40	9.15	.19
*SE*	9.47	12.70	21.91	.38
*t*/*z*	.50	.74	.41	.48
*Note.* Significant effects are indicated with an asterisk.				

## Footnotes

1 Using the Weber contrast calculation gives a value of 30% of normal contrast for the faint condition in the current study, the same calculation produces a value of 40% for the faint condition in the Sheridan and Reingold (2013) study. Using this calculation to compare across studies reveals that contrast levels between 30% to 40% of full contrast have been employed by five other studies to date (Glaholt et al., 2014; Hohenstein & Kliegl, 2014; Jainta et al., 2017; Sheridan & Reingold, 2013; Wang & Inhoff, 2010). Several other studies (for example, Liu et al., 2015) have used contrast levels much lower than this (in this case, 15% of full contrast); however, this level of contrast was deemed too low for use with older adults.

2 For sentence-level analyses, LMMs were calculated with the model structure lmer(depvar ∼ age ∗text_contrast + (1 + text_contrast|Participant) + (1 + text_contrast|Item), datafile). For critical word analyses, LMMs were calculated with the model structure lmer(depvar ∼ age ∗ text_contrast ∗ frequency + (1 + text_contrast ∗ frequency|Participant) + (1 + text_contrast ∗ frequency|Item), datafile). If a model failed to converge, the structure was pruned until convergence was achieved. The full random effects structure was used for all measures except sentence reading time in Experiment 1, and sentence reading time and rereading time in Experiment 2. For these measures, text contrast was removed from the item random effects structure. Analyses for continuous variables for both untransformed and log-transformed data produced the same patterns of results, and so only results for untransformed data are reported. Sentence-level measures for number of regressions, rereading time, and number of first-pass skips can include zero values, so a small amount (<1) was added to each zero to allow log transformation of these data.

3 Single-fixation duration, that is, the duration of the first fixation when the target received only one fixation, was also calculated. However, in all cases, results were very similar to the results for first-fixation duration, and so single-fixation duration is not reported.

4 For all sentence-level comparisons of the young versus middle-aged group, additional Bayes factor (BF) analyses were undertaken to confirm the lack of differences between these two age groups. These analyses were computed using the lmBF function within the BayesFactor package (Morey & Rouder, 2015) in R (R Core Team, 2015), with the scaling factor for g-priors set to 0.5, and using 100,000 Monte Carlo iterations. Participants and items were specified as random factors. These analyses favored a null model with no interaction for these age groups (BFs <1).

5 Hohenstein and Kliegl (2014) examined the effect of text contrast on preview benefit for young adults. They used the gaze-contingent boundary technique to compare reading times on words with accurate and inaccurate previews. However, in this study, not only was there no effect of stimulus quality on parafoveal preview benefit—there was also no effect of stimulus quality on critical word reading times. Nevertheless, these null effects should be interpreted cautiously, as text contrast was manipulated as a between-participants variable and critical words were capitalized (see General Discussion for consideration of the possible role of capitalization in mediating effects of stimulus contrast).

6 Note that the contrast of stimuli in the parafovea has been shown to modulate processing of the fixated stimulus (Levitt & Lund, 1997; Xing & Heeger, 2000).

7 The magnitude of the contrast reduction may not be equal across age groups. Further research may explore this possibility by equating contrast in the baseline (high contrast) condition across the age groups (or for individuals) depending on contrast acuity.
